# Supplementary material for: Chronic circadian disruption modulates breast cancer stemness and immune microenvironment to drive metastasis in mice
Source: Nat Commun. 2020 Jun 24;11:3193. doi: 10.1038/s41467-020-16890-6 (PMC7314789; doi:10.1038/s41467-020-16890-6)
Supplement: Supplementary file 1 — Supplementary Information [file 41467_2020_16890_MOESM1_ESM.pdf]

Supplementary Information

Chronic circadian disruption modulates breast cancer stemness and immune microenvironment to drive metastasis in mice

Hadadi et al.

Supplementary Information includes four tables and ten figures.

	Locomotor activity			
	LD (D10-D20 post implantation)		LD (D32-D46 post implantation)	
	Period (h)	Goodness of fit **r^2**	Period (h)	Goodness of fit **r^2**
1416*	24.0	0.487	24.1	0.440
1431	24.0	0.706	24.0	0.683
1432	24.0	0.683	24.0	0.706
	**LD (D10-D20 post implantation)**		**JetLag (D32-D46 post implantation)**	
	Period (h)	Goodness of fit **r^2**	Period (h)	Goodness of fit **r^2**
1429	24.0	0.757	24.5	0.564
1430	24.0	0.522	25.5	0.019
1435*	23.8	0.739	25.5	0.041

	Temperature	
	LD (D32-D46 post implantation)	
	Period (h)	Goodness of fit **r^2**
1416*	24.0	0.642
1431	24.1	0.760
1432	24.1	0.741
	**JetLag (D32-D46 post implantation)**	
	Period (h)	Goodness of fit **r^2**
1429	23.6	0.363
1430	25.6	0.062
1435*	25.3	0.163

* mice with high tumour burden

Supplementary Table 1: Period of rest/activity and core body temperature patterns of LD and JL mice. Period of locomotor activity and core body temperature pattern with respective coefficients of determination (R^2) values defined by fitting a cosine algorithm. Mice age was 15 to 16 weeks at Day (D)10-D20 and 18 to 19 weeks at D32-D46 post microchip implantation. Source data are provided as a Source Data file.

Mouse number	Experimental conditions (JL or LD)	Lung metastasis	number of primary tumours for HES	Grade	Score (1 to 4)	Tumours used for injections (see Figure 4)
1501	LD	-	4	early carcinoma	3	
				early carcinoma	3	
				adenoma	2	
				adenoma	2	
1502	LD	-	1	early carcinoma	3	
1635	JL	-	2	early carcinoma	3	
				early carcinoma	3	
1636	JL	+	3	early carcinoma	3	
				early carcinoma	3	
				adenoma	2	
1639	JL	-	1	adenoma	2	
1643	JL	+	2	late carcinoma	4	
				late carcinoma	4	
2279	LD	-	2	hyperplasia	1	YES
				adenoma	2	
2280	LD	-	2	adenoma	3	
				adenoma	3	
1129	LD	-	3	adenoma	2	YES
				adenoma	2	
				adenoma	2	
2636	LD	+	3	early carcinoma	3	YES
				late carcinoma	4	
				late carcinoma	4	
2522	JL	-	2	late carcinoma	4	YES
				late carcinoma	4	
1012	LD	+	2	early carcinoma	3	YES
				late carcinoma	4	
1013	LD	-	2	early carcinoma	3	YES
				early carcinoma	3	
1113	JL	+	2	late carcinoma	4	YES
				late carcinoma	4	
2520	JL	-	2	adenoma	2	YES
				early carcinoma	3	
2604	JL	-	1	early carcinoma	3	YES
2602	JL	+	2	late carcinoma	4	YES
				late carcinoma	4	

Supplementary Table 2. Grades of primary tumours from LD (blue) and JL (orange) mice. Grades were defined according to the PyMT tumour classification by Lin and col. (2003). Scores (1 to 4) illustrate the aggressiveness of the tumours (1-2 in green being the less aggressive and 3-4 in red the most aggressive). Tumours from mice used for the tumour initiation study (Figure 4) are highlighted in yellow. 19 primary tumours from LD mice (n=8) and 17 primary tumours from JL mice (n=9) have been analysed.

	LD		JL		
	Mean pg.ml^{-1}	SD	Mean pg.ml^{-1}	SD	p-value
MCP-1/CCL2	UDL		UDL		
KC/CXCL1	146.6 (n=7)	87.38	181.5 (n=8)	129.1	0.5573
MIP-2/CXCL2	5.754 (n=10)	3.905	5.88 (n=10)	7.995	0.9648
LIX/CXCL5	1327 (n=10)	668.1	1303 (n=10)	659.6	0.9372
SDF-1/CXCL12	255.2 (n=8)	140	377.9 (n=6)	187	0.2033
IL-1b	UDL		UDL		
IL-2	UDL		UDL		
**IL-4 ****	88.44 (n=10)	9.23	71.52 (n=10)	13.24	0.0039
IL-6	8.112 (n=5)	7.581	7.272 (n=6)	6.295	0.845
IL-10	UDL		UDL		
IL-12p70	115.9 (n=6)	75.59	171 (n=6)	160.7	0.4715
IFNγ	UDL				
G-CSF	176.7 (n=10)	180.8	200 (n=10)	168.6	0.7699
GM-CSF	UDL		UDL		
M-CSF	5.679 (n=10)	3.226	6.678 (n=10)	1.657	0.3952
TNFα	UDL		UDL		
VEGF	14.77 (n=10)	8.201	18.14 (n=10)	14.09	0.5214

Supplementary Table 3. Cytokine and chemokine quantification. Luminex technology is used to determine circulating cytokine/chemokine concentration in the plasma of JL and LD mice . UDL: under detection level. Indicated (n) represent number of biological replicates.

Antibodies	Clone	Source	Cat#
PB anti-mouse CD45	30-F11	BioLegend	103125
PB anti-mouse CD31	390	BioLegend	102421
PB anti-mouse TER-119	TER-119	Biolegend	116231
BV421 anti-mouse CD140A	APA5	BD Biosciences	566293
BV510 anti-mouse CD24	M1/69	BioLegend	101831
BV510 Rat IgG2b, κ isotype ctrl	*RTK4530*	BioLegend	400646
FITC anti-mouse/rat CD29	HMβ1-1	BioLegend	102205
FITC Armenian Hamster IgG isotype ctrl	*HTK888*	BioLegend	400905
PE anti-human/ouse CD49f	GoH3	BioLegend	313611
PE Rat IgG2a, κ Isotype ctrl	*RTK2758*	BioLegend	400507
PE/Cy7 anti-mouse/human CD44	IM7	BioLegend	103029
PE/Cy7 Rat IgG2b, κ isotype ctrl	*RTK4530*	BioLegend	400617
APC anti-mouse CD90.1 (Thy-1.1)	OX-7	BioLegend	202526
APC Mouse IgG1,κ isotype Ctrl	*MOPC-21*	BioLegend	400119
APC-Vio770 anti-mouse CD326 (EpCAM)	caa7-9G8	Miltenyi Biotec	130-102-137
APC-Vio770 Rat IgG1,κ isotype ctrl	*ES26-14D1.11*	Miltenyi Biotec	130-103-036
AF750 anti-mouse CXCR1/CD181	1122A	R&D Biotechne	FAB8628S
AF750 Rabbit IgG control	*60024B*	R&D Biotechne	IC1051S
PE-Vio770 anti-mouse CXCR2/CD182	REA942	Miltenyi Biotec	130-115-636
PE-Vio770 REA Control	*REA293*	Miltenyi Biotec	130-113-452
APC anti-mouse CXCR4/CD184	REA107	Miltenyi Biotec	130-102-245
APC REA Control	*REA293*	Miltenyi Biotec	130-113-446
FITC anti-mouse/human CD11b	M1/70	BioLegend	101205
FITC Rat IgG2b, κ isotype ctrl	*RTK4530*	BioLegend	400605
PE anti-mouse CD64 (FcγRI)	X54-5/7.1	BioLegend	139303
PE Mouse IgG1, κ isotype ctrl	*MOPC-21*	BioLegend	400111
PE/Cy7 anti-mouse CD11c	N418	BioLegend	117317
PE/Cy7 Armenian Hamster IgG isotype ctrl	*HTK888*	BioLegend	400921
APC anti-mouse I-A/I-E (MHCII)	M5/114.15.2	BioLegend	107613
APC Rat IgG2b, κ isotype ctrl	*RTK4530*	BioLegend	400611
APC-Vio770 anti-mouse Ly-6G	REA526	Miltenyi Biotec	130-107-916
APC-Vio770 REA Control	*REA293*	Miltenyi Biotec	130-104-634
PE/Dazzle 594 anti-mouse CD45	30-F11	BioLegend	103145
BV510 anti-mouse CD3	17A2	BioLegend	100233
APC/Fire750 anti-mouse CD8a	53-6.7	BioLegend	100765
APC/Fire750 Rat IgG2a, κ isotype ctrl	*RTK2758*	BioLegend	400567
AF488 anti-mouse CD4	GK1.5	BioLegend	100423
True-Nuclear Mouse Treg Flow Kit		BioLegend	320029
APC/PE anti-mouse CD4/CD25		BioLegend	78929
AF488 anti-mouse/rat/human FOXP3	150D	BioLegend	79176
AF488 Mouse IgG1,κ isotype ctrl	*MOPC-21*	BioLegend	79477
AF488 anti-PyMT		Santa Cruz	sc-53481

Supplementary Table 4. List of antibodies used for flow cytometry.

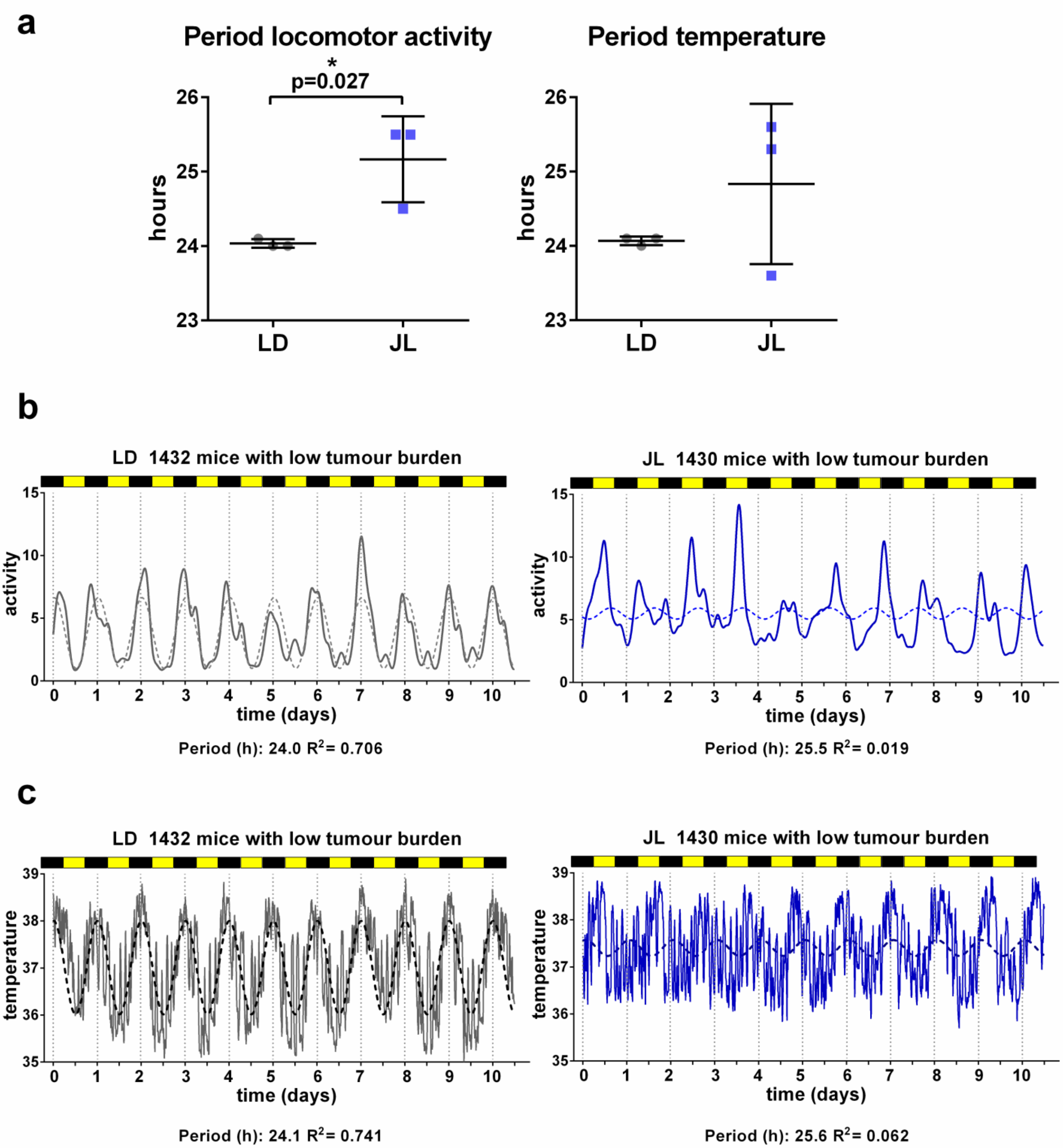

Supplementary Figure 1. Telemetry analysis. (a) Comparison of locomotor activity and temperature rhythmicity between LD (gray) (n=3) and JL (blue) (n=3) mice at age of 18 to 19 weeks. Data are presented as scatter dot plots, with lines representing the median with interquartile range (error bars). p-value calculated from an unpaired two-sided t-test. (b) Observed (solid line) vs modelled (dotted line) locomotor activity rhythmicity in LD vs JL mice with matched tumour burden (c) Observed (solid line) vs modelled (dotted line) temperature rhythmicity in LD vs JL mice with matched tumour burden. The dotted lines represent the modelled curves derived from the telemetry data using cosine algorithm. Respective period (h) and the coefficient of determination (R^2) presented for each activity and temperature patterns. Source data are provided as a Source Data file. Indicated (n) represent number of biological replicates.

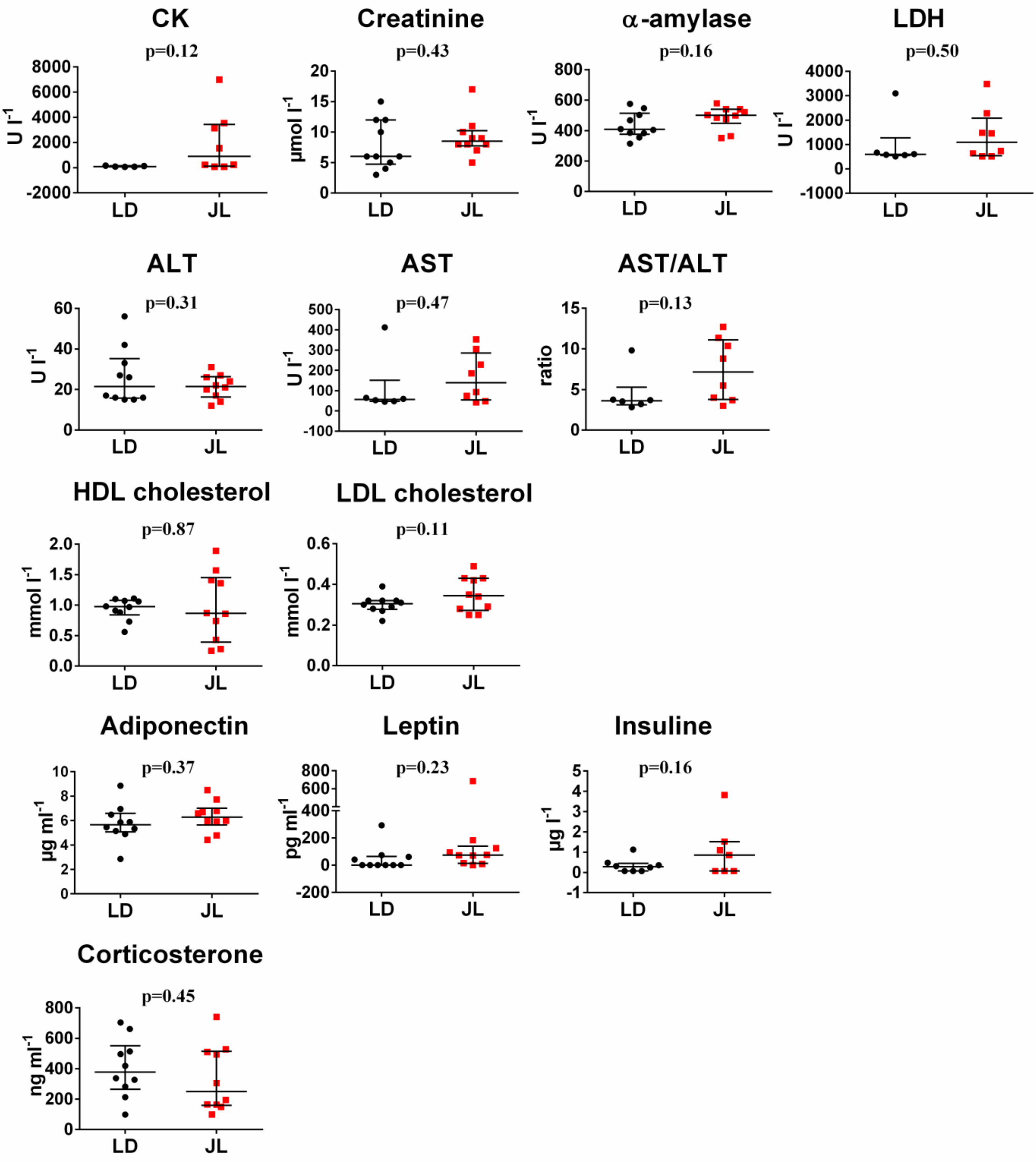

Supplementary Figure 2. Blood biochemistry and hormone levels. Quantification of creatine kinase (CK)(n=5;8), creatinine, α-amylase, lactate dehydrogenase (LDH)(n=6;8), alanine aminotransferase (ALT), aspartate aminotransferase (AST)(n=6;8), AST/ALT ratio(n=6;8), high density lipoprotein (HDL) cholesterol, low density lipoprotein (LDL) cholesterol, adiponectin, leptin(n=4;9), insulin(n=8;7), and corticosterone in LD and JL mice (n=10;10 respectively if it is not stated otherwise). Data are presented as scatter dot plots, with lines representing the median with interquartile range (error bars). p-value calculated from an unpaired two-sided t-test. Indicated (n) represent number of biological replicates. Source data are provided as a Source Data file.

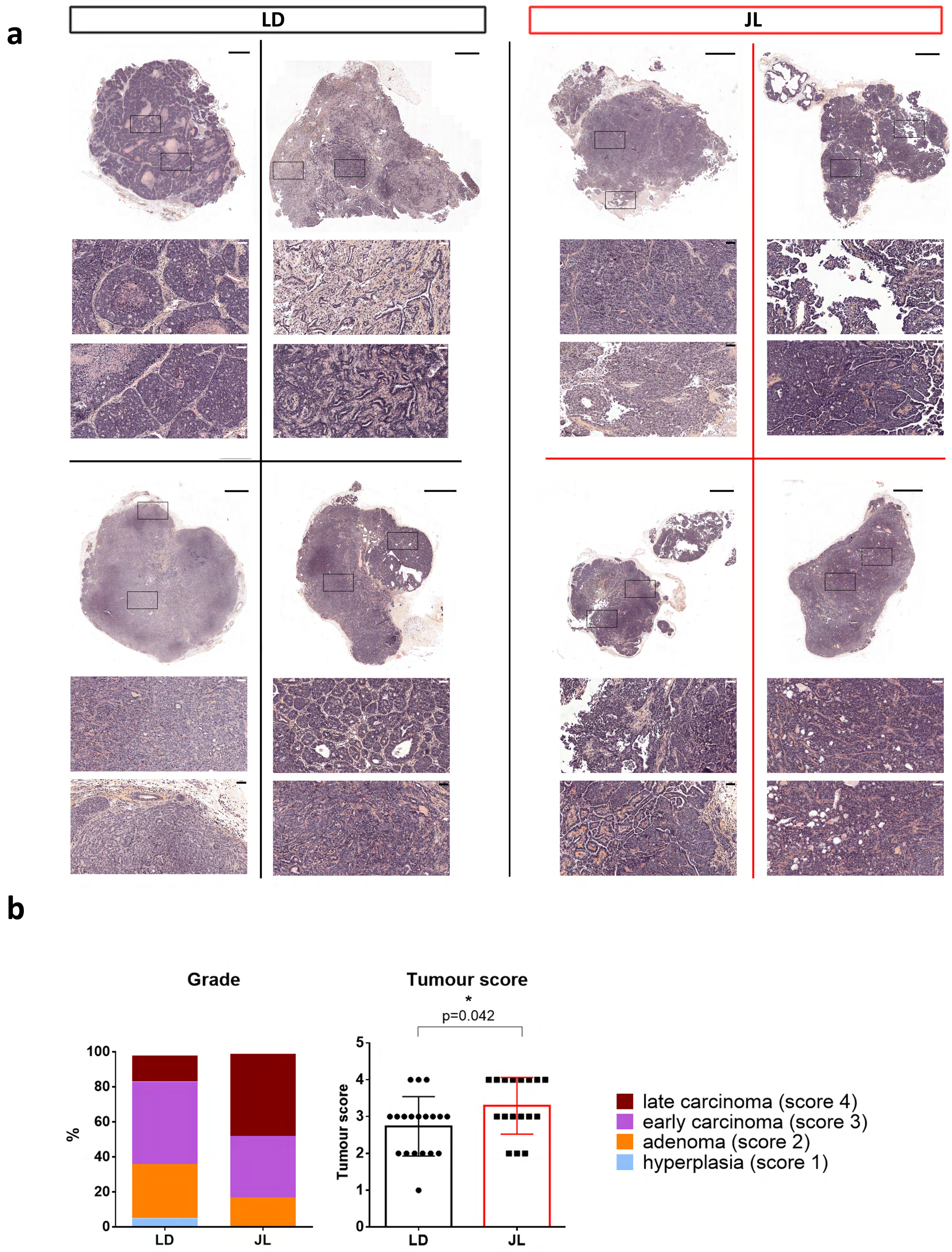

Supplementary Figure 3. Histological analysis of primary tumours. (a) A global view of eight primary tumours from LD (n=4) and JL (n=4) mice is shown, each scale bar represents 1mm. For each tumour, black squares indicate the localization of two higher magnifications for which each scale bar represents 50µm (b) Sections of primary tumours (n=19 for LD and n=17 for JL) were analysed and graded according to Lin and col. (2003), in a four-stage classification scheme that includes hyperplasia (score 1, blue), adenoma/mammary intraepithelial neoplasia (MIN) (score 2, orange), and early (score 3, violet) and late carcinoma (score 4, dark red) (see supplementary Table 2). Grade and score data presented as bar graph and scattered dot plot with bars and lines representing the mean and SD (error bars). p-value calculated from an unpaired two-sided t-test. Indicated (n) represent number of biological replicates.

a

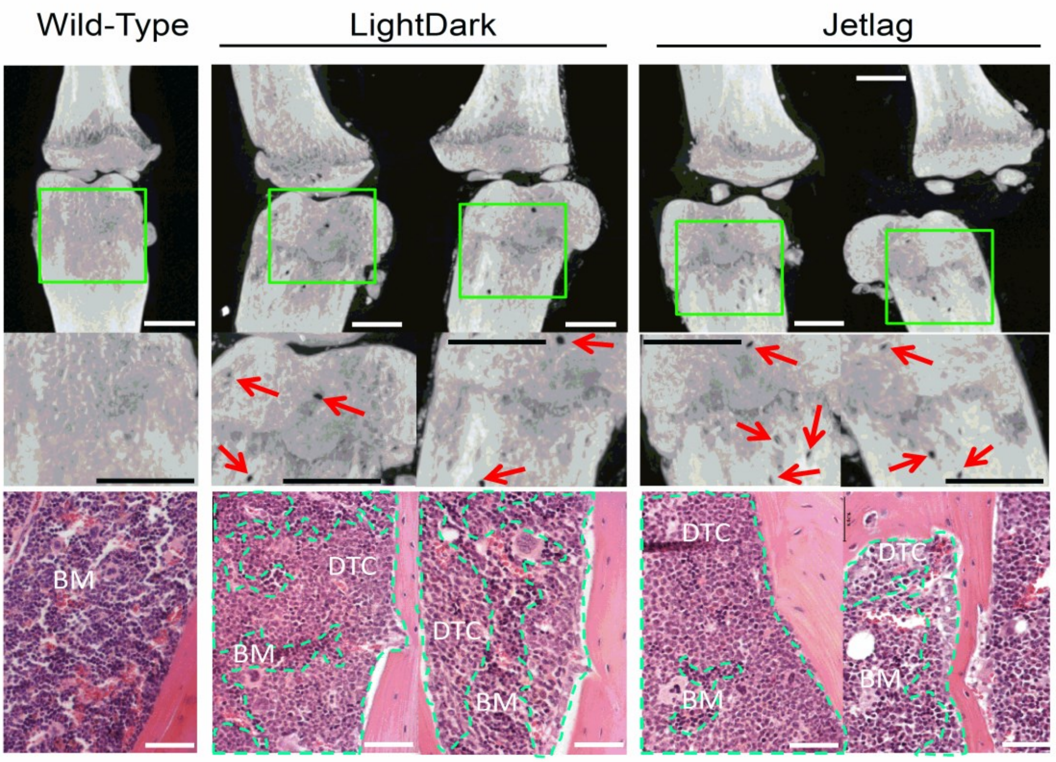

b

Distribution of metastatic foci

Conditions	number of metastatic foci		
	0	<3	>3
LD	18	6	1
JL	10	6	5

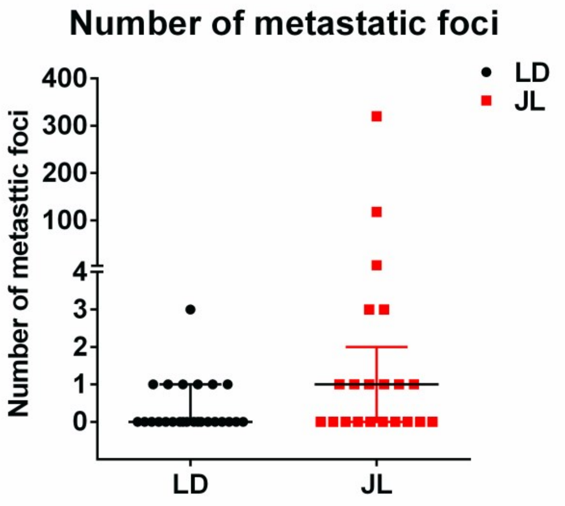

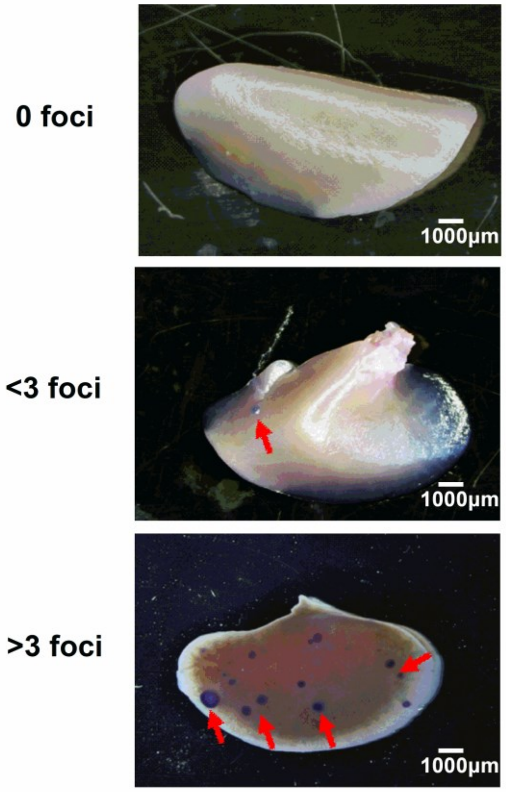

Supplementary Figure 4. Cancer cell dissemination in bones and lungs. (a) Cancer cell dissemination in bones. (Upper panel) µCT 3D reconstruction (with CTVox program). Lower images are magnifications of the green box. Each red arrow indicates abnormal tissue that could be osteolytic lesions. Each scale bar represents 1 mm. (Lower panel) H&E images representing areas, delimited by green dotted lines, containing abnormal cells that could be tumoural cells. BM: bone marrow, DTC: disseminated tumour cells. Scale represent 100µm (b) Quantification of lung metastasis. Lung metastases were detected using immunocytochemistry and revealed by NBT/BCIP staining (blue labelling, red arrows). Lung metastases were counted and lungs were classified in three categories: no metastatic foci, from 1-3 metastatic foci, and more than 3 metastatic foci. Data are presented as scatter dot plots, with lines representing the median with interquartile range (error bars).

a

Sample	Tissue	Condition	Metastasis	Total CleanReads	Total MappingRatio	Uniquely MappingRatio	Total GeneNumber
**1726-BM**	Bone Marrow	LD	M-	23865426	82.97	78.68	15002
**1726-T**	Primary Tumour	LD	M-	24124640	70.69	66.73	16137
**1734-BM**	Bone Marrow	LD	M-	23877046	82.68	78.46	14964
**1734-T**	Primary Tumour	LD	M-	24071835	72.7	69.52	16253
**1789-BM**	Bone Marrow	JL	M+	24097072	82.68	78.31	14481
**1789-T**	Primary Tumour	JL	M+	24030875	73.61	70.2	16122
**1791-BM**	Bone Marrow	JL	M+	24011010	79.74	75.55	14727
**1791-T**	Primary Tumour	JL	M+	24066011	63.7	60.91	15971
**1970-BM**	Bone Marrow	LD	M+	24022511	82.01	77.4	15126
**1970-T**	Primary Tumour	LD	M+	23939182	60.81	58.14	16373
**2066-BM**	Bone Marrow	LJ	M+	24103025	81.97	77.71	15098
**2423-BM**	Bone Marrow	LD	M-	24070346	79.89	75.98	15391
**2423-T**	Primary Tumour	LD	M-	24095531	55.54	53.04	16106
**2514-BM**	Bone Marrow	LD	M+	24103621	83.03	78.49	15073
**2514-T**	Primary Tumour	LD	M+	24125957	70.72	67.76	16517
**2521-BM**	Bone Marrow	JL	M-	24086082	80.86	76.58	14760
**2521-T**	Primary Tumour	JL	M-	24048917	72.57	69.47	16434
**2522-BM**	Bone Marrow	JL	M-	24037282	82.8	78.48	14710
**2522-T**	Primary Tumour	JL	M-	24078366	75.81	72.92	16315

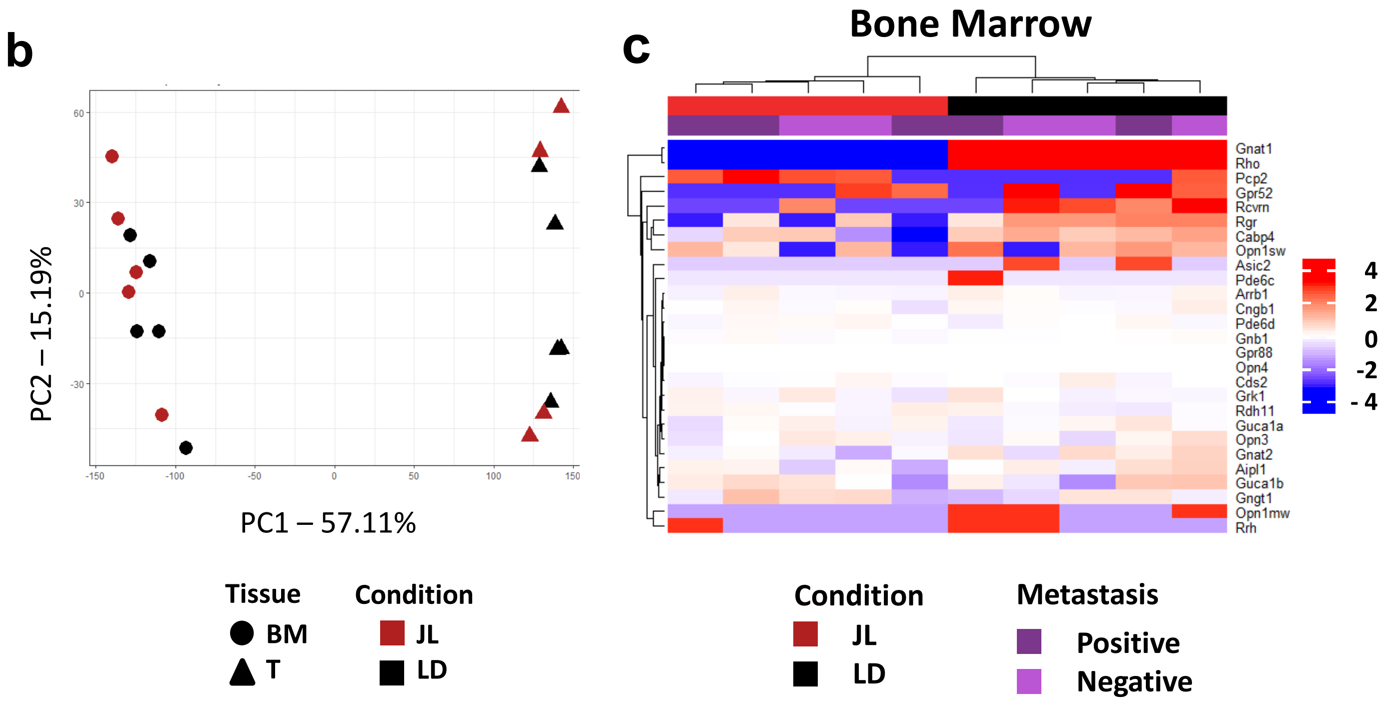

Supplementary Figure 5. Transcriptome analysis of bone marrow mono-nucleated cells and primary tumours. (a) Global statistics for read numbers and mapping of mRNA-seq data. (b) Principal Components Analysis of all samples based on the expression values of shared genes (12556 genes). JL samples (n=9) are in red and LD (n=10) in black. Bone marrow (BM) samples are represented by circles and primary tumours (T) by triangles. (c) Heatmap based on the expression of all expressed genes associated with the GO term Phototransduction (GO:0007602) in mono-nucleated cells from bone marrow. Samples appear as columns and genes as rows; samples are labelled by the experimental conditions (JL (n=5) in red and LD (n=5) in black) and the presence (dark violet)/absence (light violet) of metastasis. The colour scale represents different expression levels, red for high expression, and blue for low expression. Hierarchical clustering was performed using Euclidean distance and the Ward.D2 criterion for agglomeration. Indicated (n) represent number of biological replicates.

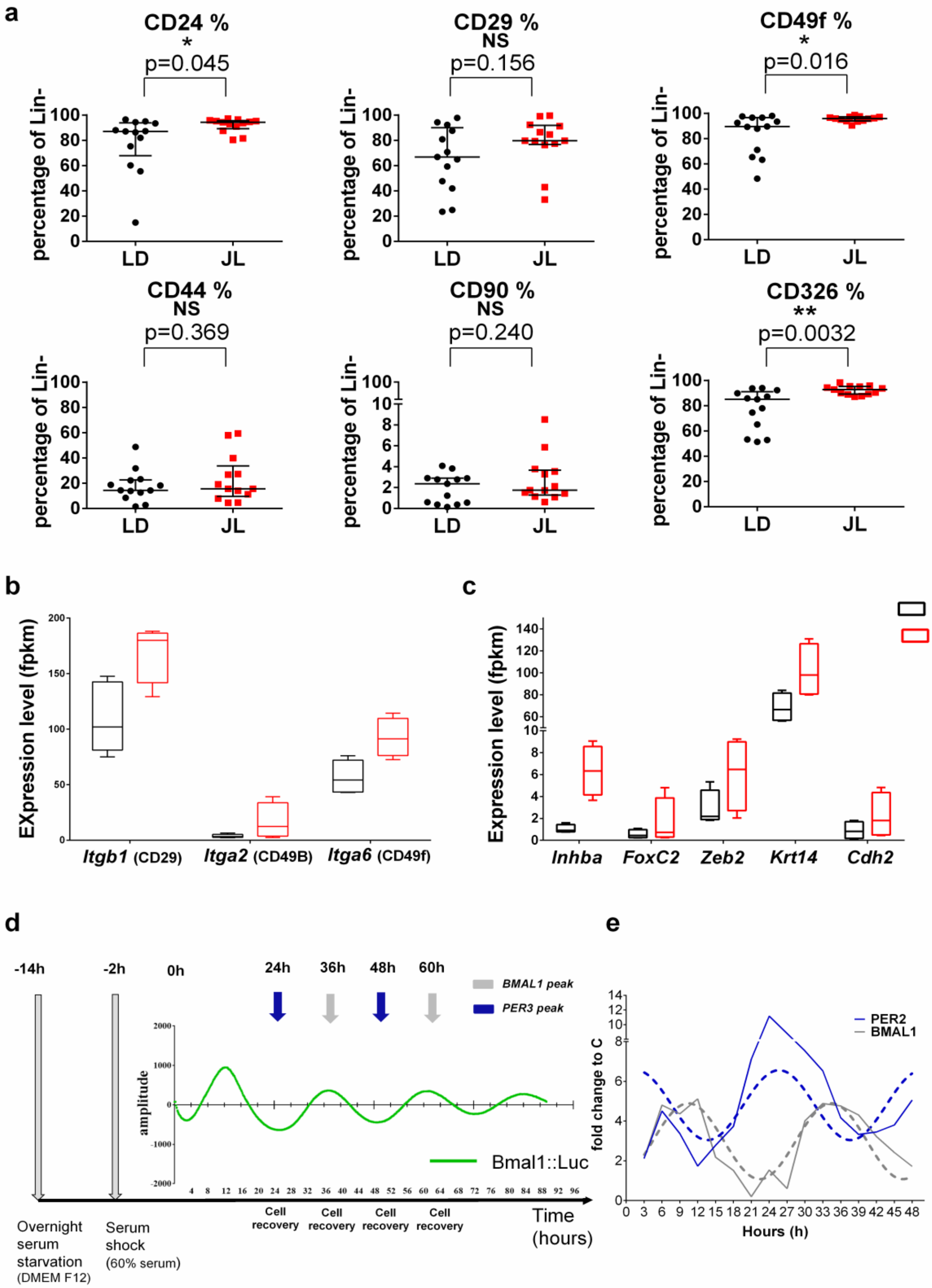

Supplementary Figure 6. Expression of stemness and EMT markers in mammary tumours cell. (a) Relative distribution of stemness markers CD24, CD29, CD49f, CD44, CD90, and CD326 in LD (n=13) and JL (n=13) Lin$^-$ (CD45$^-$CD31$^-$CD140a$^-$Ter119$^-$) tumour cells, as determined by flow cytometry. Data are shown as scatter dot plots with lines representing the median with interquartile range (error bars). p-values obtained from an unpaired two-sided t-test. (b) Gene expression values (FPKM) of *Itgb1*, *Itga2*, and *Itga6* and (c) EMT-associated genes in primary tumours from LD (black boxes, n=5) and JL (red boxes, n=4) mice. Data are presented as box-and-whisker plots. Variability is depicted using medians (line in the box), 25th and 75th percentiles (box), and min to max (whiskers). Respective log2FoldChange (log2FC) and p-values are listed in Supplementary Data 3. (d) Synchronisation protocol used on human mammary epithelial cells to study stemness at different circadian phases. *BMAL1* oscillations are represented by the green line, using a BMAL1:LUC reporter cell line. (e) Oscillatory expression profile of *PER2* and *BMAL1* in synchronised MCF12A cells. Indicated (n) represent number of biological replicates. Source data are provided as a Source Data file.

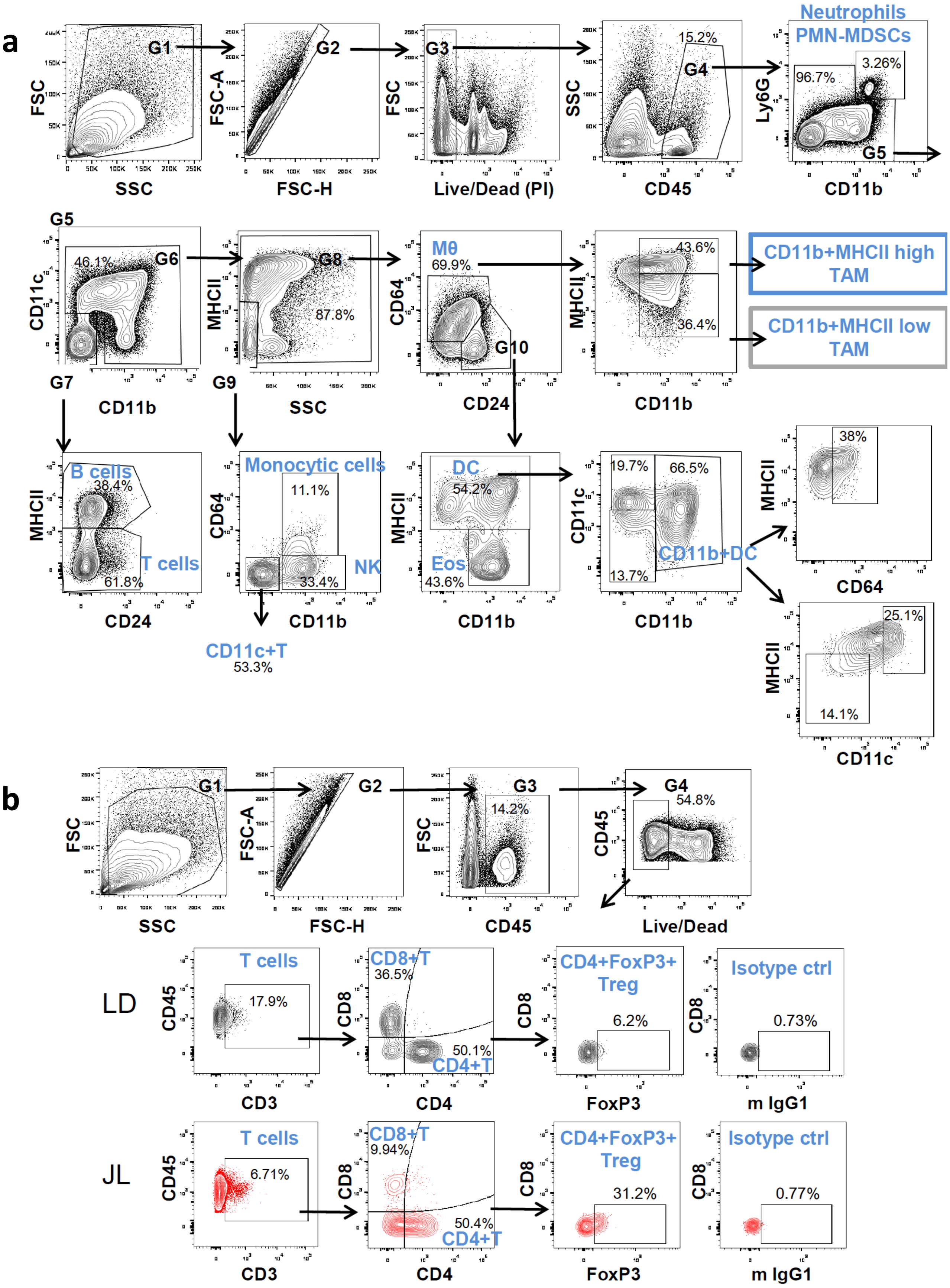

Supplementary Figure 7. Flow cytometry gating strategies. (a) Gating strategy to identify the main tumour infiltrating immune cell populations described by Yu and col. (2016). Data presented on Fig. 5a-c, Fig. 6h, Supplementary Fig. 8a and 10f. (b) Gating strategy to identify T cell populations (data presented on Fig. 5e,g, Fig. 6e, Supplementary Fig. 8b and 10a,e) with representative flow cytometry plots of intracellular FoxP3 staining to identify regulatory T cells (Treg) in primary tumours (data presented on Fig. 5g).

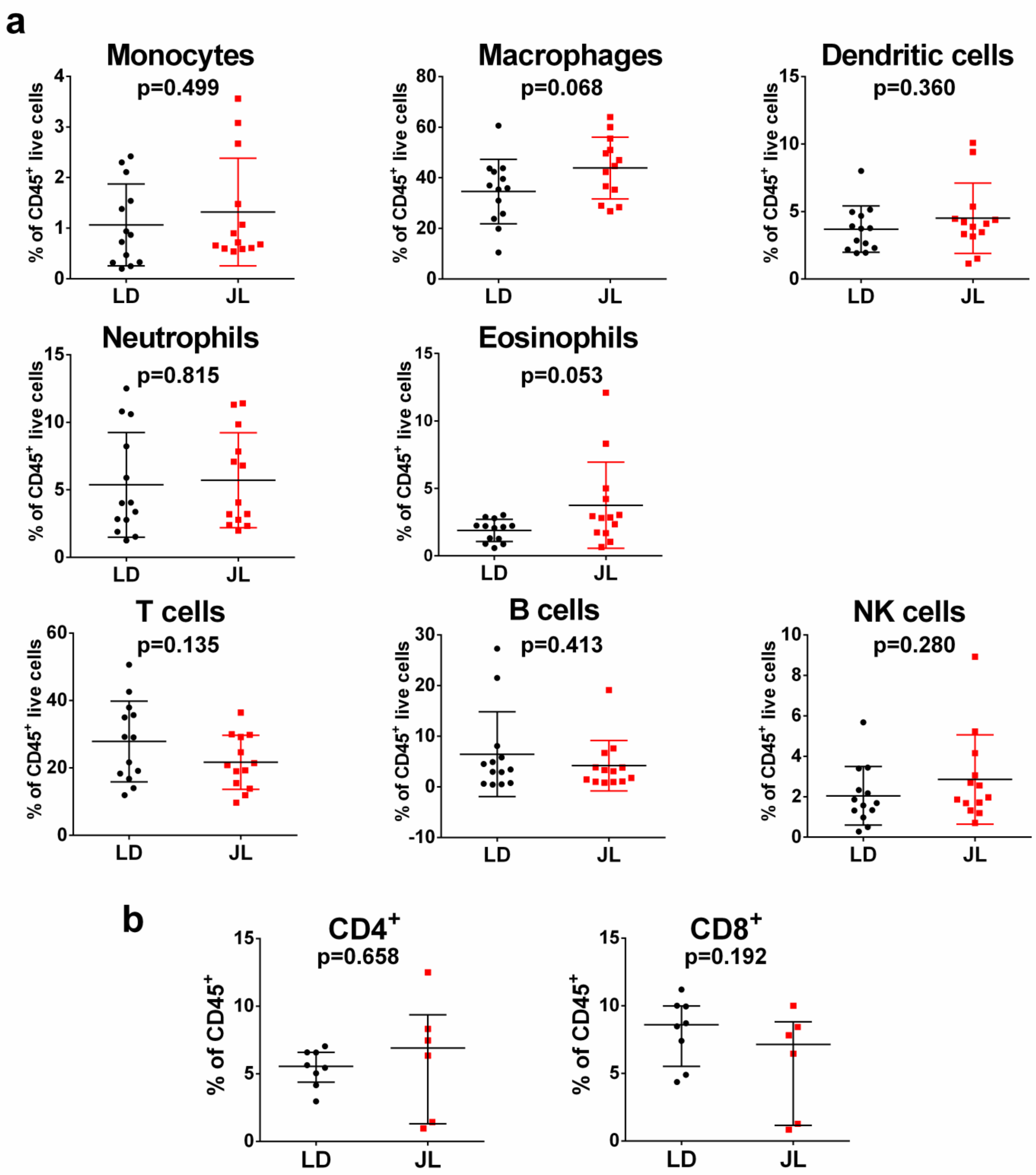

Supplementary Figure 8. Main immune cell types in tumours and peripheral blood. (a) Main immune cell types in primary tumours. Proportions of monocytes, macrophages, dendritic cells, neutrophils, eosinophils, T-cells, B-cells, and NK-cells were quantified from LD (n=13) and JL (n=13) mice. Data are presented as scatter dot plots with lines representing the median and interquartile range (error bars). p-value calculated from an unpaired two-sided t-test. (b) Peripheral blood CD4+ and CD8+ T cells in LD (n=8) and JL (n=6) mice. Data are presented as scatter dot plots with lines representing the median with interquartile range (error bars). p-value calculated from an unpaired two-sided t-test. Indicated (n) represent number of biological replicates. Source data are provided as a Source Data file.

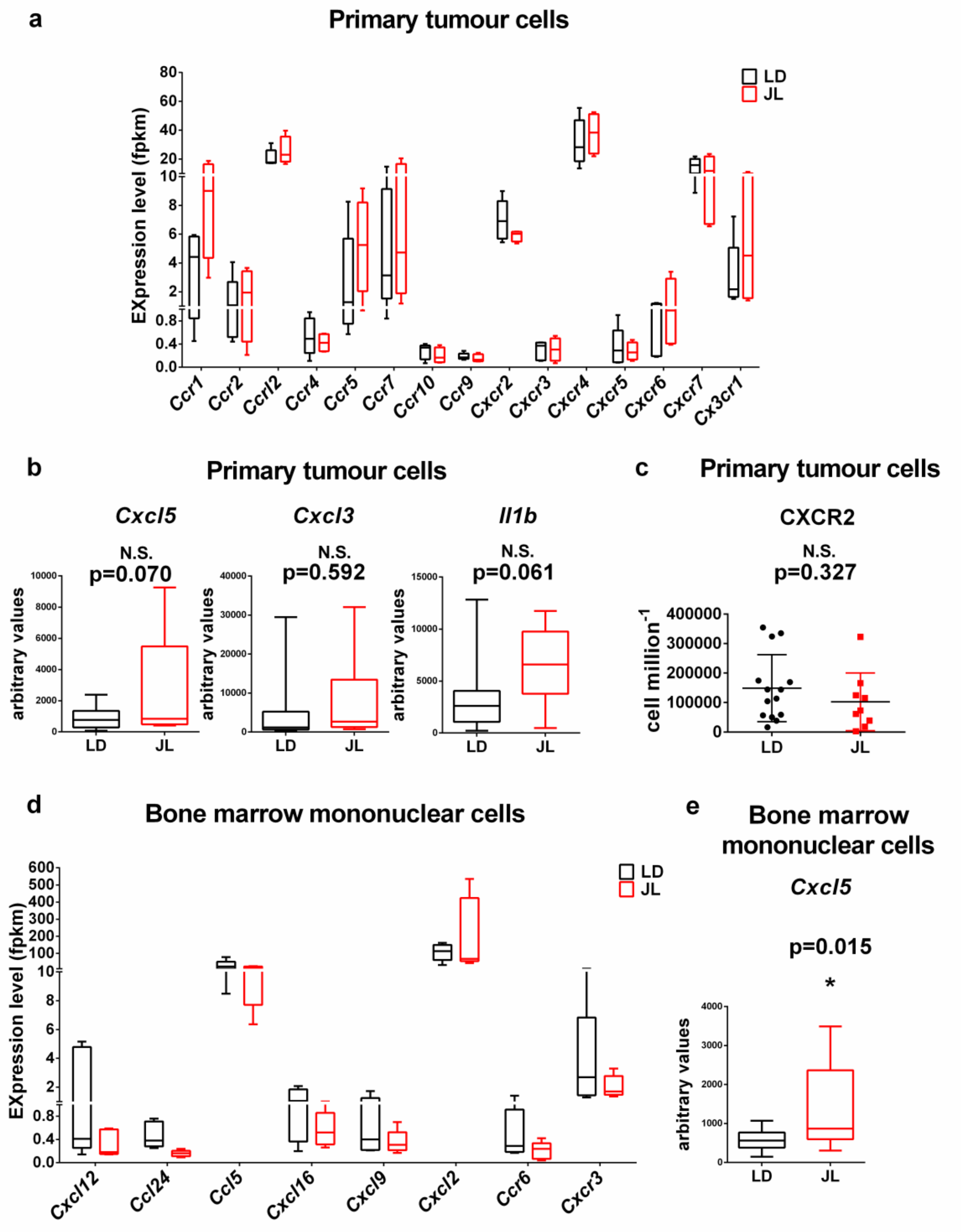

Supplementary Figure 9. Expression of chemokines/cytokines and their receptors. (a) Gene expression values (FPKM) of chemokine/cytokine receptors in primary tumours from LD (n=5) and JL (n=4) mice. Data are presented as box-and-whisker plots. Respective log2FoldChange and p-values are listed in Supplementary Data 3. (b) Expression values of *Cxcl5*, *Cxcl3*, and *Il1b* quantified by real-time PCR in primary tumour cells from LD (n=13) and JL (n=11) mice. Data are presented as box-and-whisker plots. p-value calculated from an unpaired two-side t-test. (c) Flow cytometry analysis of CXCR2 expression in cancer cells from primary tumours of LD (n=14) and JL (n=9) mice. Data are presented as scatter dot plots with lines representing the median with interquartile range (error bars). p-value calculated from an unpaired two-side t-test. (d) Gene expression values (FPKM) of the most up- or downregulated cytokines/chemokines and their receptors in bone marrow from LD (n=5) and JL (n=4) mice. Data are presented as box-and-whisker plots. Respective log2FoldChange and p-values are listed in Supplementary Data 3. (e) Expression values of *Cxcl5* quantified by real-time PCR in bone marrow cells from LD (n=13) and JL (n=11) mice. Data are presented as box-and-whisker plots. p-value calculated from an unpaired two-sided t-test. For all box-and-whisker plots, variability is depicted using medians (line in the box), 25th and 75th percentiles (box), and min to max (whiskers). Source data are provided as a Source Data file.

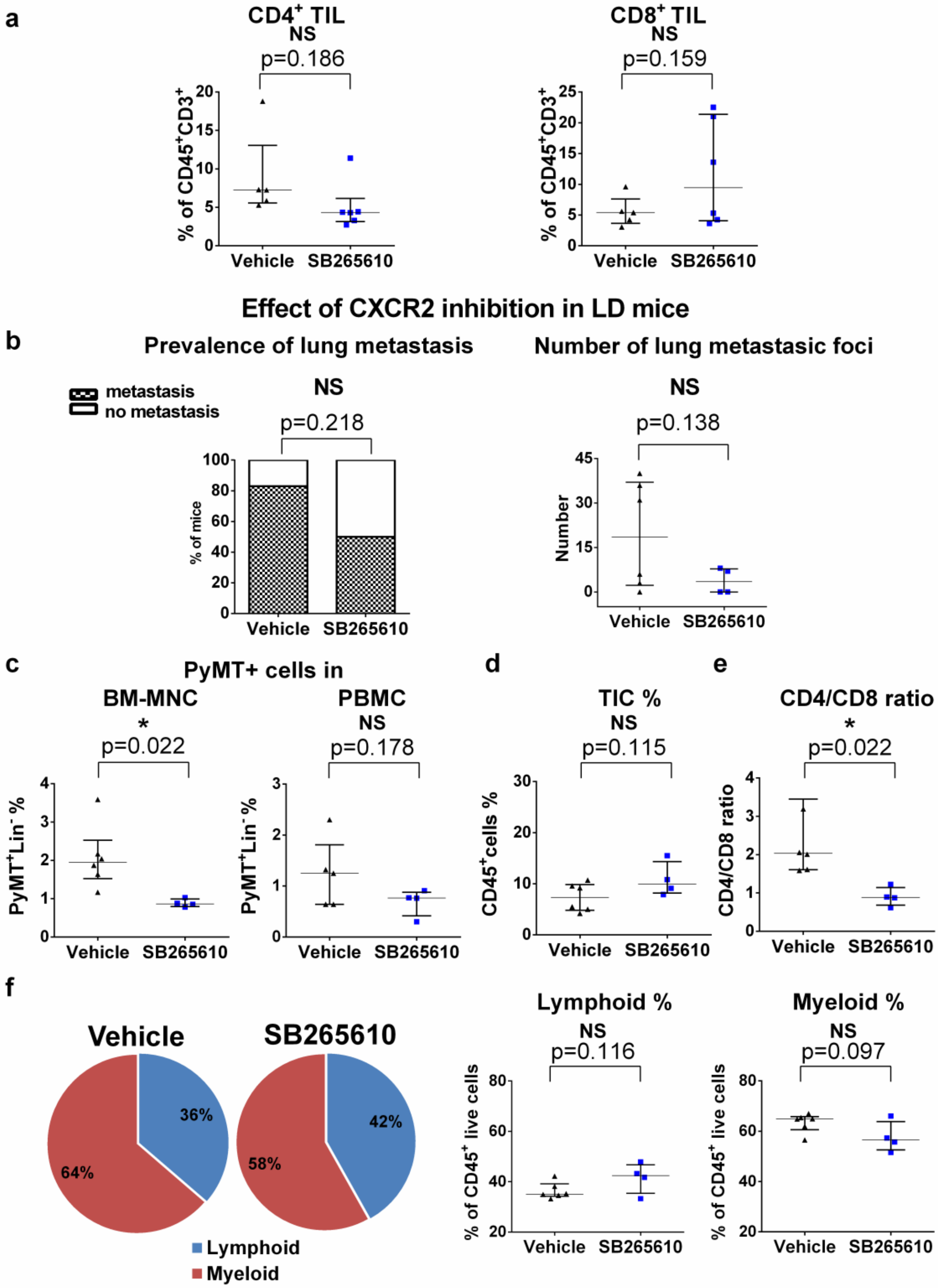

Supplementary Figure 10. Effects of CXCR2 inhibition on tumour development in JL and LD mice. (a) Tumour-infiltrating lymphocytes (TIL) in vehicle (n=5) or CXCR2 inhibitor treated (n=6) JL mice. Data are presented as scatter dot plots with lines representing the median with interquartile range (error bars). p-value calculated from an unpaired two-sided t-test. (b-f) Effects of CXCR2 inhibition in LD mice: (b) Prevalence of lung metastasis and the number of lung metastatic foci in mice injected with vehicle (n=6) or SB265610 (n=4). Data presented as bar plot and scatter dot plot with lines representing median with interquartile range (error bars), p-value obtained from binomial two-sided test and unpaired two-sided t-test. (c) Percentage of disseminated tumour cells in BM-MNC in vehicle (n=6) and SB265610 (n=4) group and in blood (n=5, 4) respectively. (d) Percentage of tumour infiltrating immune cells (TIC) in vehicle (n=6) and SB265610 (n=4) cohort. (e) CD4/CD8 ratio in tumours from vehicle (n=6) and SB265640 (n=4) groups. Data are presented as scatter dot plot with lines representing median with interquartile range (error bars). p-values are calculated from an unpaired two-sided t-test. (f) Relative distribution of lymphoid and myeloid compartment of TIC in both cohorts. Data presented as pie charts displaying the mean values of mice and as scatter dot plot with lines representing median with interquartile range (error bars). p-values are calculated from an unpaired two-sided t-test. Indicated (n) represent number of biological replicates. Source data are provided as a Source Data file.

References

1. Yu, Y.-R. A. *et al.* A Protocol for the Comprehensive Flow Cytometric Analysis of Immune Cells in Normal and Inflamed Murine Non-Lymphoid Tissues. *PLOS ONE* **11**, e0150606 (2016).
