# Supplementary material for: Chronic circadian disruption modulates breast cancer stemness and immune microenvironment to drive metastasis in mice
Source: Nat Commun. 2020 Jun 24;11:3193. doi: 10.1038/s41467-020-16890-6 (PMC7314789; doi:10.1038/s41467-020-16890-6)
Supplement: Supplementary file 3 — Description of Additional Supplementary Files [file 41467_2020_16890_MOESM3_ESM.docx]

**Description of Additional Supplementary Files**

**File name:** Supplementary Data 1

**Description:** Differentially expressed genes in Lin- bone marrow cells between LD (n=5) and JL (n=5) conditions. P-value and adjusted p-value were calculated using DESeq2 and the Wald test based on the negative binomial distribution (see Methods).

**File name:** Supplementary Data 2

**Description:** Differentially expressed genes in cancer cells from primary tumours between LD (n=5) and JL (n=4) conditions. P-value and adjusted p-value were calculated using DESeq2 and the Wald test based on the negative binomial distribution (see Methods).

**File name:** Supplementary Data 3

**Description:** Mean mRNA expression levels of highlighted genes in LD and JL mice. Data are presented as mean FPKM (Fragments Per Kilobase Million) with respective log2FoldChange (log2FC) and p-values. p-values of FPKM based analyses are obtained by unpaired two-sided t-tests. The table also includes mean expression values, log2FC and pvalues computed by DESeq2 using Wald test based on the negative binomial distribution. The second sheet includes metadata on the samples used for this gene-expression study. All the other sheets (19 in total) contain FPKM values for each sample.
